# The association between dietary vitamin B2 intake and constipation: A cross-sectional study based on NHANES 2009 to 2010

**DOI:** 10.1097/MD.0000000000045238

**Published:** 2025-10-10

**Authors:** Fengwei Liu, Siyu Liu, Jia Chen, Jiamei Fu

**Affiliations:** a Heilongjiang University of Chinese Medicine, Harbin, China; b The First Affiliated Hospital of Heilongjiang University of Chinese Medicine, Harbin, China.

**Keywords:** constipation, cross-sectional study, National Health and Nutrition Examination Survey, vitamin B2

## Abstract

Previous studies have established a correlation between dietary vitamin intake and constipation. However, research specifically addressing the relationship between vitamin B2 and constipation is scarce. This study aims to explore the link between chronic constipation and dietary vitamin B2 intake among adults participating in the National Health and Nutrition Examination Survey. Data from 5134 participants aged 20 years and older, collected during the 2009 to 2010 National Health and Nutrition Examination Survey, were analyzed. Dietary information was gathered through a 24-hour dietary recall. Participants who reported constipation “always,” “most of the time,” or “sometimes” in the past 12 months were categorized as having constipation. The relationship between dietary vitamin B2 intake and chronic constipation was assessed through various statistical methods, including multivariate logistic regression, restricted cubic spline regression, subgroup analysis, curve-fitting, and inflection point analysis. The adjusted multivariate logistic regression model indicated an association between vitamin B2 intake and a reduced risk of constipation (OR = 0.78, 95% CI: 0.71–0.87, *P* < .001). Restricted cubic spline regression revealed a nonlinear relationship between vitamin B2 intake and constipation risk. Inflection point analysis determined that the risk of chronic constipation decreased as vitamin B2 intake increased up to a daily intake of 1.849 mg, with this decrease being statistically significant (*P* < .01). Above this threshold, the association was not statistically significant. This study demonstrates a negative correlation between dietary vitamin B2 intake and chronic constipation. Specifically, an increase in vitamin B2 intake up to a certain level appears to reduce the risk of chronic constipation. However, the role of vitamin B2 intake beyond this threshold requires further investigation.

## 1. Introduction

Constipation is a common chronic gastrointestinal disorder characterized by difficulties in defecation, such as infrequent stools and a feeling of incomplete evacuation.^[1]^ It affects 15% to 20% of the global population and is more prevalent in females, with their rates being approximately double those of males.^[2]^ The modern lifestyle and dietary habits have led to a significant increase in functional constipation, imposing a greater burden on healthcare systems.^[3]^ Chronic constipation is associated with diminished quality of life due to heightened levels of anxiety and depression.^[4]^ Moreover, it significantly increases the risk of colorectal and other digestive cancers, thereby endangering patient health and survival.^[5]^ Sedentary lifestyles, low-fiber diets, and diabetes are recognized as high-risk factors for constipation. Addressing these controllable factors through dietary changes and lifestyle adjustments is crucial.^[6]^ Recent calls for large-scale studies aim to identify potential risk factors for constipation, facilitating improvements in lifestyle to mitigate constipation risk.^[7]^

B vitamins (B1, thiamin; B2, riboflavin; B3, niacin; B5, pantothenic acid; B6, pyridoxine; B7, biotin; B9, folate; and B12, cobalamin) are essential micronutrients for all cellular life. These vitamins are critical trace elements for human physiological metabolism and regulatory processes, serving as cofactors for numerous enzymes in the body. However, most B vitamins must be obtained through dietary intake because they cannot be synthesized by the human body.^[8]^ An insufficient supply of B vitamins impairs the nervous and digestive systems.^[9]^ A common clinical symptom in the affected digestive system is abnormal defecation. For instance, insufficient vitamin B1 may lead to softer stools and increased intestinal peristalsis.^[10]^ Vitamin B2, or riboflavin, is essential as it can be converted into coenzymes that facilitate energy production from carbohydrates, fats, and proteins. Deficiency in riboflavin may induce endoplasmic reticulum stress and apoptosis, potentially disrupting normal physiological functions.^[11]^ Vitamin B2 also exhibits antioxidant and anti-inflammatory properties^[12]^ and is being explored for its potential in cancer therapy.^[13]^ In summary, vitamin B2 is vital for maintaining human health. Despite some studies linking dietary micronutrient intake, including vitamin B2, to chronic constipation,^[14]^ large-scale research on this relationship remains limited. This study aims to clarify the association between dietary vitamin B2 intake and persistent constipation in the general population.

## 2. Materials and methods

### 2.1. Survey description

National Health and Nutrition Examination Survey (NHANES) is a comprehensive, cross-sectional study conducted by the National Center for Health Statistics of the Centers for Disease Control and Prevention. It collects health-related data from a representative sample of the U.S. population using a stratified, multistage sampling design. NHANES aims to assess the health and nutritional status of American adults and children. The survey was approved by the research ethics review board of the National Center for Health Statistics, and all participants provided written informed consent.

### 2.2. Study population

**Figure 1.**
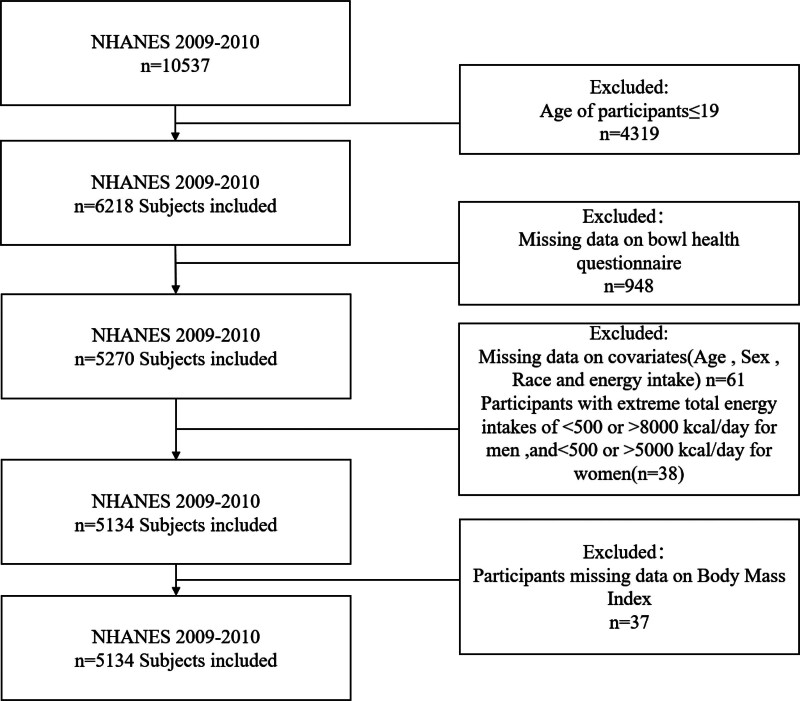
Flowchart of participant selection form NHANES 2009 to 2010. NHANES = National Health and Nutrition Examination Survey.

Data from the 2009 to 2010 NHANES were used to analyze intestinal health information, involving 10,537 participants after initial data extraction. A series of exclusion criteria were applied to target adults aged 20 years or older, enhancing data reliability. These criteria excluded participants under 19 years old (n = 4319), those with incomplete intestinal health data (n = 948), missing covariate data (age, sex, race, energy intake) (n = 61), individuals with extreme diets (males n = 11, females n = 27), and those missing body mass index (BMI) data (n = 37). After adjusting for missing energy information, no additional exclusions were necessary. Consequently, 5134 participants (2588 females and 2546 males) were included in further analyses, with Figure 1 illustrating the screening process.

### 2.3. Definition of constipation

The 2009 to 2010 Bowel Health Questionnaire is commonly utilized to assess chronic constipation in study objects.^[10]^ It includes the question, “How often have you been constipated in the past 12 months?” Participants responding “always,” “most of the time,” or “sometimes” are considered to have constipation, whereas those answering “rarely” or “never” are classified as non-constipated.

### 2.4. Vitamin B2 intake

Dietary intake data were gathered by estimating the consumption of energy, nutrients, and other food components from food and beverages consumed in the previous 24 hours. Participants underwent 2 24-hour dietary recall interviews; the first was conducted at a mobile examination center, and the second via telephone communication 3 to 10 days later. The average intake of vitamin B2 was calculated when participants completed both 24-hour recalls. However, if only the first recall was completed, only that data was used. The total population included in the study (n = 5134) was evenly divided into 4 groups according to vitamin B2 intake, with each group comprising 1283 participants. The vitamin B2 intake ranges defined were Q1 (0.079–1.358 mg), Q2 (1.358–1.849 mg), Q3 (1.849–2.499 mg), and Q4 (2.499–16.30 mg). Under these conditions, we were able to analyze the association between different levels of vitamin B2 intake and constipation prevalence.

### 2.5. Covariates

Numerous covariates were included in the study to mitigate the impact of potential confounding factors on the results. These covariates comprised age, gender, race, marital status, smoking status, alcohol consumption, BMI, and intakes of various dietary elements (energy, protein, carbohydrates, total sugars, dietary fiber, total fat, saturated fatty acids, monounsaturated fatty acids, polyunsaturated fatty acids, caffeine, folate, sodium, potassium, phosphorus, niacin, vitamin B1, and vitamin B6). Race was categorized into Mexican Americans, non-Hispanic Whites, non-Hispanic Blacks, and other. Marital status was divided into 3 groups: married or cohabiting, divorced, separated, or widowed, and never married. Based on smoking habits, participants were classified as never smokers (those who have never smoked or smoked fewer than 100 cigarettes in their lifetime), current smokers (those who have smoked at least 100 cigarettes and continue to smoke), and former smokers (those who have smoked at least 100 cigarettes but do not currently smoke). Participants consuming at least 12 alcoholic beverages annually were considered alcohol drinkers. Other dietary elements were treated as continuous variables.

### 2.6. Statistical analyses

Descriptive analyses were performed on all participants. Statistical analyses were conducted using R software (version 4.1.3), Empower Stats (version 6.0; X&Y Solutions, Boston), and STATA 18.0 (StataCorp LLC, College Station), with *P* < .05 denoting statistical significance. Participants with incomplete covariate data were excluded. Continuous data were analyzed using mean, standard deviation, or interquartile range, and described as standard deviation ± weighted mean due to varying data natures. Categorical variables were presented as percentages and compared using the chi-square test. Multivariate logistic regression analyses were conducted to explore the association between vitamin B2 consumption and chronic constipation. Model 1 was not adjusted; Model 2 was adjusted for age, sex, and race; Model 3 included adjustments for age, sex, race, marital status, BMI, alcohol consumption, smoking status, protein, caffeine intake, fiber, carbohydrate, total fat, total saturated fatty acids, total monounsaturated fatty acids, total polyunsaturated fatty acids, folic acid, phosphorus, sodium, potassium, niacin, and vitamin B1. Subgroup analyses were conducted to assess the effects of age, gender, race, marital status, BMI, alcohol consumption, and smoking status on outcomes. The association between vitamin B2 intake and chronic constipation was further assessed using a restricted cubic spline fitting curve. Considering the complex sampling design used by NHANES, appropriate weights were applied according to NHANES guidelines. The data were meticulously weighted to ensure accurate and nationally representative estimates.

## 3. Results

### 3.1. Baseline characteristics of participants

Table 1 presents the baseline characteristics of the study population by vitamin B2 intake. A total of 5134 participants who met the screening criteria were included in the study, including 1540 constipated individuals. Dietary vitamin B2 intake was categorized into 4 groups to summarize the population’s overall characteristics. It is noted in Table 1 that the incidence of constipation decreased progressively with increasing vitamin B2 intake.

**Table 1:** Baseline characteristics of the study population according to the vitamin B2 intake in NHANES 2009 to 2010.

Characteristics	Vitamin B2 intake (mg)					*P* value
	Total (N = 5134)	Q1 (0.079, 1.358), N = 1284	Q2 (1.358, 1.849), N = 1283	Q3 (1.849, 2.499), N = 1283	Q4 (2.499, 16.3), N = 1284	
Gender						<.001
Male	49.33 (47.35–51.31)	33.72 (30.13–37.51)	39.98 (36.09–44.01)	51.06 (47.09–54.96)	66.71 (63.05–70.26)	
Female	50.67 (48.69–52.65)	66.28 (62.49–69.87)	60.02 (56.00–63.91)	48.94 (45.04–52.91)	33.29 (29.74–36.95)	
Age	46.71 ± 16.56	45.26 ± 17.95	48.06 ± 16.32	47.13 ± 16.38	46.25 ± 15.77	<.001
20–45 yr	49.08 (47.10–51.05)	54.34 (50.51–58.10)	44.54 (40.58–48.57)	47.93 (44.00–51.88)	50.08 (46.24–53.93)	
46–65 yr	35.66 (33.72–37.66)	27.91 (24.67–31.40)	39.27 (35.24–43.46)	37.31 (33.45–41.33)	36.75 (32.96–40.71)	
65 + yr	15.26 (14.17–16.43)	17.75 (15.29–20.52)	16.19 (13.97–18.69)	14.77 (12.73–17.06)	13.16 (11.29–15.29)	
Race						<.001
Mexican American	8.63 (7.90–9.42)	12.13 (10.37–14.14)	9.50 (8.01–11.22)	7.99 (6.63–9.56)	5.98 (4.89–7.30)	
Non-Hispanic White	68.35 (66.70–69.96)	49.79 (45.92–53.65)	64.43 (60.79–67.91)	74.11 (71.04–76.87)	79.61 (77.01–82.07)	
Non-Hispanic Black	11.54 (10.61–12.53)	21.72 (19.10–24.59)	12.44 (10.53–14.65)	8.03 (6.61–9.72)	6.73 (5.48–8.25)	
Other races	11.48 (10.36–12.70)	16.36 (13.68–19.44)	13.63 (11.11–16.61)	9.93 (8.05–12.18)	7.62 (6.01–9.62)	
Marital status						<.001
Married or with partner	63.47 (61.57–65.33)	57.47 (53.62–61.24)	64.68 (60.81–68.36)	64.59 (60.79–68.26)	65.77 (62.04–69.28)	
Single or divorced	17.82 (16.47–19.26)	19.60 (16.91–22.59)	18.88 (16.15–21.95)	18.08 (15.37–21.12)	15.42 (13.09–18.10)	
Unmarried	18.71 (17.17–20.35)	22.93 (19.70–26.50)	16.44 (13.63–19.70)	17.33 (14.47–20.60)	18.81 (15.86–22.20)	
Smoking status						<.001
Yes	20.00 (18.51–21.58)	21.99 (18.97–25.35)	19.46 (16.49–22.81)	18.08 (15.30–21.22)	20.83 (18.02–23.98)	
Former smoking	24.98 (23.29–26.75)	19.38 (16.52–22.59)	23.30 (20.10–26.84)	25.64 (22.31–29.24)	29.81 (26.36–33.53)	
No	55.02 (53.05–56.97)	58.63 (54.79–62.37)	57.24 (53.22–61.17)	56.28 (52.37–60.17)	49.36 (45.49–53.18)	
Alcohol consumption						<.001
Yes	78.28 (20.22–23.30)	68.10 (64.52–71.48)	77.48 (74.20–80.45)	78.90 (75.56–81.91)	85.74 (83.02–88.09)	
No	21.72 (20.22–23.30)	31.90 (28.52–35.48)	22.52 (19.55–25.80)	21.09 (18.09–24.44)	14.26 (11.91–16.98)	
Constipation						<.001
Yes	26.84 (25.17–28.57)	37.33 (33.63–41.18)	28.54 (25.07–32.27)	23.19 (20.13–26.53)	21.30 (18.50–24.42)	
No	73.16 (71.42–74.83)	62.67 (58.82–66.37)	71.46 (67.73–74.93)	76.83 (73.47–79.87)	78.69 (75.58–81.50)	
Fiber (g)	17.34 ± 9.32	11.78 ± 6.25	15.22 ± 6.98	18.23 ± 7.88	22.28 ± 11.19	<.001
Energy intake (kcal)	2124.11 ± 835.03	1464.81 ± 462.49	1842.14 ± 545.85	2206.01 ± 635.27	2757.42 ± 931.51	<.001
Protein intake (g)	83.68 ± 35.47	55.59 ± 19.51	72.76 ± 22.77	85.55 ± 25.29	111.32 ± 40.67	<.001
Carbohydrate (g)	257.49 ± 106.02	188.59 ± 72.04	225.80 ± 81.26	265.62 ± 85.51	325.93 ± 119.01	<.001
Total fat intake (g)	79.24 ± 38.99	51.49 ± 20.87	68.07 ± 26.04	82.83 ± 30.55	105.17 ± 47.05	<.001
Total sugars (g)	114.63 ± 65.94	85.27 ± 52.10	101.08 ± 57.68	115.04 ± 57.95	146.74 ± 74.21	<.001
Caffeine intake (mg)	174.02 ± 197.03	88.56 ± 88.89	134.52 ± 129.73	177.14 ± 162.78	265.67 ± 275.88	<.001
BMI (kg/m^2^)	28.90 ± 6.70	29.06 ± 7.40	29.27 ± 6.93	28.78 ± 6.32	28.60 ± 6.31	.052
Total saturated fatty acids (g)	25.81 ± 14.08	15.75 ± 6.93	21.83 ± 8.85	26.68 ± 10.87	35.58 ± 17.32	<.001
Total monounsaturated fatty acids (g)	28.48 ± 14.82	18.69 ± 8.15	24.36 ± 9.95	29.75 ± 11.87	37.79 ± 18.27	<.001
Total polyunsaturated fatty acids (g)	17.72 ± 9.86	12.28 ± 6.49	15.56 ± 7.95	18.86 ± 8.63	22.38 ± 11.77	<.001
Folic acid (g)	419.70 ± 214.89	251.68 ± 101.94	342.17 ± 120.64	431.86 ± 147.88	594.05 ± 255.60	<.001
Sodium (mg)	3594.17 ± 1537.08	2531.82 ± 1030.67	3191.69 ± 1115.22	3703.22 ± 1197.81	4593.68 ± 1774.01	<.001
Potassium (mg)	2782.45 ± 1132.08	1800.47 ± 632.50	2407.96 ± 662.90	2860.23 ± 769.14	3730.28 ± 1245.51	<.001
Phosphorus (mg)	1415.32 ± 580.14	880.39 ± 265.23	1186.95 ± 302.63	1450.68 ± 355.97	1958.39 ± 619.86	<.001
Niacin (mg)	26.16 ± 12.43	16.92 ± 6.62	21.78 ± 7.62	26.22 ± 8.36	36.41 ± 14.54	<.001
Vitamin B1 (mg)	1.68 ± 0.81	1.029 ± 0.41	1.38 ± 0.46	1.68 ± 0.48	2.40 ± 0.94	<.001
Vitamin B6 (mg)	2.14 ± 1.22	1.29 ± 0.55	1.73 ± 0.66	2.14 ± 0.77	3.08 ± 1.59	<.001

Values are weighted mean ± SD or weighted % (95% confidence interval).

BMI = body mass index, SD = standard deviation.

Table 2 presents the characteristics of individuals in the constipation and non-constipation groups. The constipation group consisted of 67.45% females and 32.55% males, with female prevalence approximately double that of males. Among racial groups, non-Hispanic Whites exhibited higher constipation prevalence compared to other races. Additionally, constipation prevalence was greater among participants who were married or with a partner than in other groups.

**Table 2:** Baseline characteristics of the constipation group versus the non-constipation group.

Characteristics	Total adults (N = 5134)	Constipation (N = 1540)	Non-constipation (N = 3594)	*P* value
Gender				<.001
Male	49.33 (47.35–51.31)	32.55 (29.28–35.98)	55.49 (53.15–57.80)	
Female	50.67 (48.69–52.65)	67.45 (64.02–70.71)	44.51 (42.20–46.85)	
Age	46.71 ± 16.56	46.82 ± 16.73	46.67 ± 16.50	.767
20–45 yr	49.08 (47.10–51.05)	50.12 (46.49–53.75)	48.69 (46.35–51.04)	
46–65 yr	35.66 (33.72–37.66)	34.43 (30.97–38.08)	36.11 (33.79–38.49)	
65 + yr	15.26 (14.17–16.43)	15.44 (13.55–17.54)	15.20 (13.89–16.61)	
Race				<.001
Mexican American	8.63 (7.90–9.42)	10.74 (9.23–12.46)	7.86 (7.05–8.75)	
Non-Hispanic White	68.35 (66.70–69.96)	61.32 (57.97–64.57)	70.94 (69.04–72.76)	
Non-Hispanic Black	11.54 (10.61–12.53)	14.57 (12.63–16.75)	10.43 (9.40–11.54)	
Other races	11.48 (10.36–12.70)	13.37 (11.18–15.91)	10.78 (9.51–12.20)	
Marital status				.009
Married or with partner	63.47 (61.57–65.33)	63.76 (60.31–67.08)	63.36 (61.09–65.58)	
Single or divorced	17.82 (16.47–19.26)	19.79 (17.33–22.51)	17.1 (15.51–18.81)	
Unmarried	18.71 (17.17–20.35)	16.44 (13.90–19.35)	19.54 (17.68–21.53)	
Alcohol consumption				<.001
Yes	78.28 (20.22–23.30)	71.40 (68.06–74.53)	80.80 (79.03–82.46)	
No	21.72 (20.22–23.30)	28.60 (25.47–31.94)	19.20 (17.54–20.97)	
Smoking status				.003
Yes	20.00 (18.51–21.58)	22.98 (20.00–26.26)	18.91 (17.22–20.73)	
Former smoking	24.98 (23.29–26.75)	22.94 (20.08–26.07)	25.73 (23.69–27.87)	
No	55.02 (53.05–56.97)	54.08 (50.44–57.68)	55.36 (53.02–57.68)	
Fiber (g)	17.34 ± 9.32	16.48 ± 8.87	17.66 ± 9.46	<.001
Energy intake (kcal)	2124.11 ± 835.03	1966.68 ± 804.45	2181.87 ± 838.59	<.001
Protein intake (g)	83.68 ± 35.47	76.80 ± 34.62	86.21 ± 35.44	<.001
Carbohydrate (g)	257.49 ± 106.02	245.22 ± 104.69	262.00 ± 106.14	<.001
Total fat intake (g)	79.24 ± 38.99	73.04 ± 37.39	81.51 ± 39.32	<.001
Total sugars (g)	114.63 ± 65.94	111.05 ± 65.77	115.95 ± 65.95	.018
Caffeine intake (mg)	174.02 ± 197.03	159.56 ± 206.01	179.33 ± 193.36	.001
BMI (kg/m^2^)	28.90 ± 6.70	28.62 ± 6.77	29.01 ± 6.67	.068
Total saturated fatty acids (g)	25.81 ± 14.08	23.63 ± 13.19	26.61 ± 14.31	<.001
Total monounsaturated fatty acids (g)	28.48 ± 14.82	25.98 ± 13.77	29.40 ± 15.09	<.001
Total polyunsaturated fatty acids (g)	17.72 ± 9.86	16.75 ± 10.19	18.08 ± 9.71	<.001
Folic acid (g)	419.70 ± 214.89	385.74 ± 205.62	432.16 ± 216.87	<.001
Sodium (mg)	3594.17 ± 1537.08	3340.41 ± 1500.20	3687.26 ± 1539.95	<.001
Potassium (mg)	2782.45 ± 1132.08	2603.61 ± 1124.39	2848.06 ± 1127.80	<.001
Phosphorus (mg)	1415.32 ± 580.14	1309.33 ± 560.54	1454.21 ± 582.34	<.001
Niacin (mg)	26.16 ± 12.43	23.63 ± 11.78	27.08 ± 12.53	<.001
Vitamin B1 (mg)	1.68 ± 0.81	1.56 ± 0.79	1.73 ± 0.81	<.001
Vitamin B2 (mg)	2.16 ± 1.07	1.98 ± 1.04	2.23 ± 1.07	<.001
Vitamin B6 (mg)	2.14 ± 1.22	1.95 ± 1.21	2.20 ± 1.22	<.001

Values are weighted mean ± SD or weighted % (95% confidence interval).

BMI = body mass index, SD = standard deviation.

Table 3 demonstrates the univariate logistic regression analyzing the relationship between various variables and constipation. Being female and non-Hispanic White were identified as risk factors for constipation. Dietary factors such as higher intakes of energy, protein, carbohydrates, total fat, saturated fatty acids, monounsaturated fatty acids, folate, sodium, phosphorus, and potassium were also linked to increased constipation. Conversely, alcohol consumption was suggested as a potential protective factor against constipation.

**Table 3:** Univariate logistic regression of the association between all variables and constipation.

	Constipation, non-constipation	*P* value
Gender		
Male	–	–
Female	2.58 (2.15–3.09)	<.001
Age		
20–45 yr	–	–
46–65 yr	0.93 (0.76–1.13)	.458
65 + yr	0.99 (0.81–1.21)	.900
Race		
Mexican American	–	–
Non-Hispanic White	0.63 (0.51–0.79)	<.001
Non-Hispanic Black	1.02 (0.79–1.32)	.866
Other races	0.91 (0.68–1.22)	.514
Marital status		
Married or with partner	–	–
Single or divorced	1.15 (0.94–1.41)	.185
Unmarried	0.84 (0.66–1.06)	.145
Smoking status		
Yes	1.24 (0.99–1.55)	.052
Former smoking	0.91 (0.74–1.13)	.396
No	–	–
Alcohol consumption		
Yes	0.59 (0.49–0.72)	<.001
No	–	–
Fiber (g)	0.98 (0.97–0.99)	.007
Energy intake (kcal)	0.99 (0.99–0.99)	<.001
Protein intake (g)	0.99 (0.98–0.99)	<.001
Carbohydrate (g)	0.99 (0.99–0.99)	.001
Total fat intake (g)	0.99 (0.99–0.99)	<.001
Total sugars (g)	0.99 (0.99–0.99)	.093
Caffeine intake (mg)	0.99 (0.99–1.00)	.079
BMI (kg/m^2^)	0.99 (0.97–1.00)	.186
Total saturated fatty acids (g)	0.98 (0.97–0.99)	<.001
Total monounsaturated fatty acids (g)	0.98 (0.97–0.99)	<.001
Total polyunsaturated fatty acids (g)	0.98 (0.97–0.99)	.012
Folic acid (g)	0.99 (0.99–0.99)	<.001
Sodium (mg)	0.99 (0.99–0.99)	<.001
Potassium (mg)	0.99 (0.99–0.99)	<.001
Phosphorus (mg)	0.99 (0.99–0.99)	<.001
Niacin (mg)	0.97 (0.96–0.98)	<.001
Vitamin B1 (mg)	0.75 (0.66–0.85)	<.001
Vitamin B6 (mg)	0.82 (0.74–0.90)	<.001

OR are displayed with their 95% confidence intervals and *P*-value.

BMI = body mass index.

### 3.2. Associations between vitamin B2 intake and risk of constipation

Table 4 displays the logistic regression analyzing the association between dietary vitamin B2 intake and constipation. Vitamin B2 intake was inversely associated with constipation risk (OR = 0.78, 95% CI: 0.71–0.87, *P* < .001). Three models were used for analysis, with ORs, 95% confidence intervals, and *P*-values reported. Bold values indicate a significance level of *P* < .05. Model 1 was not adjusted; Model 2 was adjusted for age, sex, and race; Model 3 included adjustments for age, sex, race, marital status, BMI, alcohol consumption, smoking status, protein, caffeine intake, fiber, carbohydrate, total fat, total saturated fatty acids, total monounsaturated fatty acids, total polyunsaturated fatty acids, folic acid, phosphorus, sodium, potassium, niacin, and vitamin B1.

**Table 4:** Logistic regression of the association between vitamin B2 intake and constipation.

Characteristic	Constipation, non-constipation	*P* value
Model 1		
Continuous (vitamin B2)	0.78 (0.71–0.87)	**<.001**
Q1 (0.079–1.358 mg)	–	
Q2 (1.358–1.849 mg)	0.67 (0.53–0.85)	**.001**
Q3 (1.849–2.499 mg)	0.51 (0.39–0.64)	**<.001**
Q4 (2.499–16.3 mg)	0.45 (0.36–0.58)	**<.001**
Model 2		
Continuous (vitamin B2)	0.92 (0.83–1.02)	.112
Q1 (0.079–1.358 mg)	–	
Q2 (1.358–1.849 mg)	0.74 (0.58–0.94)	**.015**
Q3 (1.849–2.499 mg)	0.63 (0.49–0.81)	**<.001**
Q4 (2.499–16.3 mg)	0.61 (0.41–0.76)	**.002**
Model 3		
Continuous (vitamin B2)	0.98 (0.82–1.19)	.896
Q1 (0.079–1.358 mg)	–	
Q2 (1.358–1.849 mg)	0.74 (0.57–0.94)	**.021**
Q3 (1.849–2.499 mg)	0.62 (0.46–0.83)	**.001**
Q4 (2.499–16.3 mg)	0.58 (0.44–0.79)	**.045**

Bold values indicate a significance level of *P* < .05.

OR are displayed with their 95% confidence intervals and *P*-value.

Model 1 was not adjusted; Model 2 was adjusted for age, sex, and race; Model 3 included adjustments for age, sex, race, marital status, BMI, alcohol consumption, smoking status, protein, caffeine intake, fiber, carbohydrate, total fat, total saturated fatty acids, total monounsaturated fatty acids, total polyunsaturated fatty acids, folic acid, phosphorus, sodium, potassium, niacin, and vitamin B1.

BMI = body mass index.

### 3.3. Subgroup analyses

**Figure 2.**
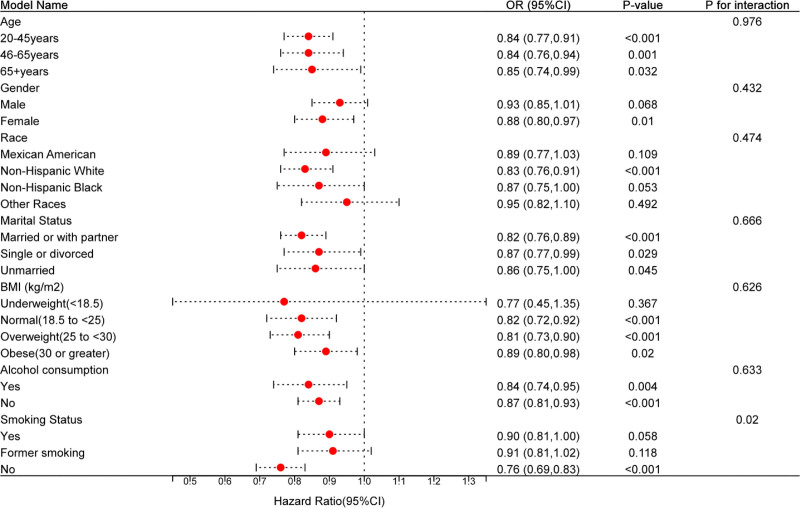
Subgroup analyses of the association between dietary vitamin B2 intake and constipation.

Figure 2 shows subgroup analyses conducted to explore the association between constipation and dietary vitamin B2 intake. The interaction *P*-value was > .05, indicating no significant interaction among groups.

### 3.4. Linear relationship between dietary vitamin B2 intake and constipation

**Figure 3.**
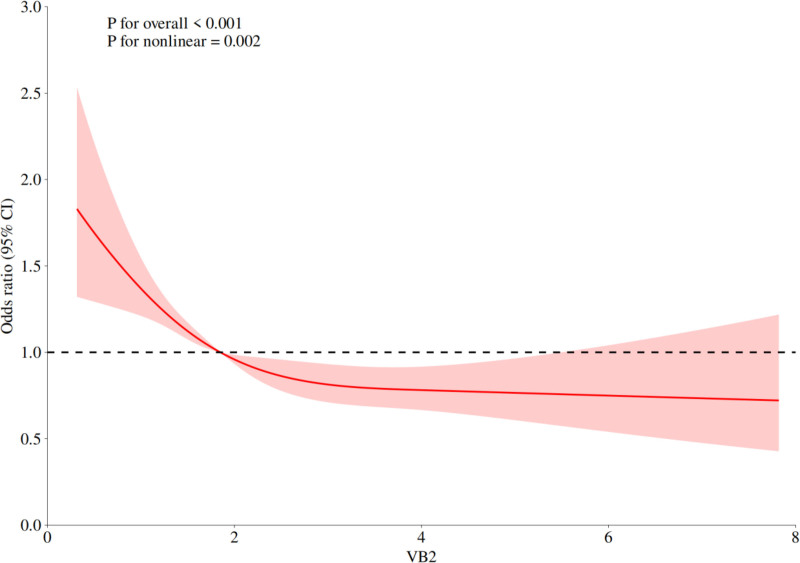
The negative correlation between vitamin B2 and constipation using RCS modeling. RCS = restricted cubic spline.

Figure 3 illustrates the negative correlation between vitamin B2 and constipation using restricted cubic spline modeling. A curve was fitted to demonstrate a nonlinear relationship between vitamin B2 intake and chronic constipation.

Table 5 displays the inflection point analysis of vitamin B2 intake and chronic constipation.

**Table 5:** Vitamin B2 intake and chronic constipation inflection point analysis.

	OR (95% CI)	*P* value
Vitamin B2 < 1.849 (mg)	0.57 (0.42–0.78)	<.001
Vitamin B2 ≥ 1.849 (mg)	0.96 (0.84–1.11)	.600

OR are displayed with their 95% confidence intervals and *P*-value.

With a daily intake of 1.849 mg identified as the inflection point, the analysis showed that the risk of chronic constipation decreased as vitamin B2 intake increased up to 1.849 mg, with statistically significant differences (*P* < .01). However, no statistically significant differences were observed in the risk of chronic constipation when the daily intake exceeded 1.849 mg.

## 4. Discussion

Dietary vitamin B2 was found to be negatively correlated with constipation within a certain range, with increased intake of vitamin B2 reducing the risk after accounting for confounding factors. It is notable that the NHANES Bowel Questionnaire BHQ060, based on the Bristol Stool Scale, is commonly used to define constipation.^[14]^ In this study, constipation was defined using the NHANES Bowel Questionnaire BHD050, as most clinical patients self-report their condition, focusing on self-perceived symptoms. Analyses have shown that self-reported symptoms result in a higher reported incidence of constipation compared to other diagnostic criteria.^[15]^ Further analyses of the NHANES database revealed a negative association between vitamin B6 intake and constipation prevalence, with vitamin B6 enhancing intestinal motility and stool softening.^[16]^ Vitamin E may alleviate constipation through its antioxidant properties and regulation of intestinal microbiota.^[17]^ Vitamin D supplementation is suggested to improve intestinal motility disorders and relieve constipation.^[18]^ A deficiency in vitamin B2 is linked to gastrointestinal symptoms.^[19]^ Additionally, various transporter proteins are crucial in the absorption and excretion of riboflavin and have been suggested as biomarkers for breast cancer resistance protein activity.^[20]^ Fluorescein, a byproduct of riboflavin’s photodegradation, may influence intestinal bacteria.^[21]^ Psychological factors are also implicated in inducing constipation. Studies linking B vitamins with depression’s immunometabolism and the gut–brain axis indicate that a reduced state of depression can stimulate the production of beneficial substances.^[22]^ Riboflavin metabolites may positively affect tissue repair following intestinal inflammation.^[23]^ However, establishing a causal relationship between vitamin B2 and constipation is challenging due to the cross-sectional nature of the NHANES database and potential recall bias. Confounding factors related to constipation may not be comprehensively included due to limitations inherent to the NHANES data. Dietary trace element intake is estimated in the questionnaire, introducing potential data bias. Nonetheless, results from the NHANES database are considered more reliable due to its representation of a large, national sample. This article detailed the association between vitamin B2 and constipation to aid future research on this relationship.

## 5. Conclusion

In conclusion, an association between vitamin B2 and a reduced risk of constipation was confirmed in the study. Therefore, optimal vitamin B2 intake should be considered to promote a healthier diet and reduce the incidence of constipation. Additionally, the underlying mechanisms by which vitamin B2 intake improves constipation require further investigation.

## Acknowledgments

We thank the participants of the NHANES and the NHANES staff. We thank Heilongjiang Provincial Undergraduate Colleges and Universities Central Government supports the reform and development fund projects of local universities for providing financial support.

## Author contributions

**Conceptualization:** Jiamei Fu.

**Data curation:** Fengwei Liu, Siyu Liu.

**Formal analysis:** Fengwei Liu, Jia Chen.

**Investigation:** Fengwei Liu, Jia Chen.

**Methodology:** Fengwei Liu, Jia Chen, Jiamei Fu.

**Project administration:** Fengwei Liu, Siyu Liu, Jiamei Fu.

**Resources:** Siyu Liu.

**Software:** Siyu Liu, Jia Chen.

**Supervision:** Fengwei Liu, Siyu Liu, Jia Chen.

**Validation:** Siyu Liu, Jia Chen.

**Visualization:** Siyu Liu, Jia Chen.

**Writing – original draft:** Fengwei Liu, Jiamei Fu.

**Writing – review & editing:** Fengwei Liu, Jiamei Fu.
